# Status quo of the public’s knowledge of probiotics based on video-sharing platforms

**DOI:** 10.1186/s12889-023-15456-7

**Published:** 2023-03-28

**Authors:** Chun-Hui Jiang, Jia-Jia Xu, Chao Xu, Shi-Yue Chen, Jia-Yun Chen, Jing-Song Xia, Zhuan Liao, Wen-Bin Zou, Xue Fang

**Affiliations:** 1 Department of Gastroenterology, Changhai Hospital, the Naval Military Medical University, 168 Changhai Road, Shanghai, 200433 China; 2 Department of General Medicine, Beicai Community Health Service Center of Pudong New District, Shanghai, China; 3 Department of Radiology, Changhai Hospital, the Naval Military Medical University, Shanghai, China; 4 School of Health Science and Engineering, the University of Shanghai for Science and Technology, Shanghai, China

**Keywords:** Probiotics, The public, Knowledge, Online videos, Social media

## Abstract

**Background:**

Probiotics have been deemed multipotent and unprecedentedly applied in the health field recently. However, there are challenges in promoting credible and reliable resources while avoiding misinformation regarding probiotics for the public.

**Methods:**

This study analysed 400 eligible probiotic-related videos selected from YouTube, and the three most popular video-sharing platforms (Bilibili, Weibo and TikTok) in China. Video retrieval was performed on September 5^th^, 2022. GQS and tailored DISCERN tool assess each video’s quality, usage, and reliability. A comparative analysis of videos from different sources was carried out.

**Results:**

The identity distribution of probiotic video-producers was predominantly experts (*n* = 202, 50.50%), followed by amateurs (*n* = 161, 40.25%) and health-related institutions (*n* = 37, 9.25%). The videos’ content category mainly discussed the function of probiotics (*n* = 120, 30%), the way to choose suitable products (*n* = 81, 20.25%), and the methods for taking probiotics (*n* = 71, 17.75%).The overall quality of videos was moderate (3/5 point) assessed by GQS, while the usage (1/6 point) and reliability (2/5 point) detailing probiotics assessed by tailored DISCERN tool were poor. The attitude of probiotic video-producers was primarily positive (*n* = 323, 80.75%), followed by neutral (*n* = 52, 13.00%) and negative (*n* = 25, 6.25%) (*P* < 0.001).

**Conclusions:**

The current study showed that videos on social media platforms publicise important information including the concepts, usage, and precautions of probiotics to the public. But the overall quality of uploaded videos about probiotics was unsatisfactory. More efforts are needed to improve the higher-quality content of probiotic-related online videos and better propagate probiotic knowledge to the public in the future.

**Supplementary Information:**

The online version contains supplementary material available at 10.1186/s12889-023-15456-7.

## Introduction

Probiotics are live microorganisms, attracting much attention for promoting health and well-being. Probiotics can balance the disturbed gut microbiome, modulate gastrointestinal disorders, enhance immunity, relieve stress, improve metabolism, and exert other effects [1]. Therefore, probiotics successfully exist worldwide as consumed food supplements and incubate a growing multi-billion-dollar industry [2, 3]. Foods, such as yogurt, snacks, and infant formulas, are embellished with probiotics, let alone probiotics-targeted medicine [4, 5]. The public is increasingly using probiotics for better health or following doctors’ recommendations for illness [6, 7]. However, there are still many issues regarding the safety and efficacy of probiotics, improper market regulation, and inadequate public knowledge about probiotics.

Social media are an essential vehicle for acquiring knowledge, disseminating information, exchanging experiences, and sharing opinions [8]. It is more evident during the COVID-19 pandemic because epidemic prevention caused decreased access to medical resources or voluntary avoidance of the healthcare system by the public [9, 10]. Due to social media’s strong interaction and socialisation, people, especially those with chronic illness, will be significantly affected during healthcare decisions [11]. Social media use dynamically increased by 20–80% worldwide during the pandemic crisis [12]. Social media platforms are crucial in providing instructions on healthcare procedures such as cardiopulmonary resuscitation [13]. They also facilitate the earlier detection and better management of disease states such as arrhythmias and heart failure by disseminating relevant medical knowledge [14]. However, social media is also a carrier of misleading or inaccurate information, which can pose a health risk to viewers [12, 15]. Previous studies showed that the average quality of YouTube material on health-related information was poor (2.68/5-point) by the Global Quality Scale (GQS), while the average quality of TikTok, Bilibli, and Weibo were rated poor or very poor (36.56/75-point) by the DISCERN scoring system [16, 17]. Therefore, it is critical to investigate the veracity and credibility of probiotics-related videos on online social media platforms.

Currently, no study has been identified that analyses the status quo of probiotic videos online, and the public’s knowledge of probiotics. The current study analyses online probiotic videos to investigate the quality of social media on probiotics and the knowledge of the public regarding probiotics. The study provides valuable information for researchers, manufacturers, and regulators to improve probiotic research, education, and development, enhance the quality of probiotic videos online, and promote the publicity and rational clinical usage of probiotics.

## Materials and methods

### Search strategy

Video retrieval was performed on a single day, September 5^th^, 2022, to reduce the bias incurred by newly uploaded videos. Videos were found using the search interfaces of YouTube™ website (www.youtube.com), Bilibili (www.bilibili.com), Weibo website (https://weibo.com), and TikTok (Chinese version: www.douyin.com). The search term was set as “Probiotics”. The search history on each platform was deleted immediately before searching. Each web page was scrolled down until it reached the bottom of the page, and the entire page was recorded as an HTML. All the videos were recorded as a recording screen for further analysis. According to the comprehensive sorting rank calculated by each video-sharing platform, we watched videos one by one from high-rank to low-rank.

Two investigators (Xu JJ and Xu C) independently viewed and assessed all videos. Any discrepancies between investigators were resolved by discussion with a third author (Chen SY) for consensus.

### Videos selection criteria

The inclusion criteria for targeted videos were: 1) videos in the Chinese and English language only; 2) videos reporting any aspect of Probiotics, but not limited to definition, application, personal experience, product evaluation, beneficial or side effects. The exclusion criteria of videos were: 1) duplicates; 2) non-relevant; 3) advertisements; 4) probiotics used for animals; 5) non-intestinal related probiotics such as probiotic toothpaste, shampoo, cosmetics, and other derivatives; 6) videos in languages other than Chinese and English. Finally, the first 100 videos on each platform that met inclusion criteria were selected for further analysis [18].

### Collection of video features

Each video was separately searched on four online platforms to gather the following information: date of upload, clip length, the identity of video producers (experts, health-related institutions, or amateurs), the attitude of video producers (positive, neutral, or negative), and the number of positive/negative remarks over the total remarks by audiences and main content.

Video producers’ identities were classified as experts, amateurs, and health-related institutions. Experts referred to those who were medical staff, professionals, and probiotics-related researchers. Amateurs referred to those who were individuals without medical and probiotics-related backgrounds. Health-related institutions included medical company, universities, media, and online education organizations.

Content category analyses were mainly extracted considering six aspects as follows: 1) introduction about probiotics; 2) function related to the probiotics; 3) methods to choose suitable probiotics; 4) doses and frequency of using probiotics; 5) assessment on the probiotic-related food or products; and 6) precautions in using probiotics.

### Assessment of each video

Tools from prior studies were incorporated, including GQS [19], as well as the usage and reliability scores tailored from the DISCERN tool (Quality Criteria for Consumer Health information) [20, 21], to evaluate each video and related online platform.

All videos were analysed for the usage, reliability, and quality of information based on point scales. Usage assessment of video content was scored from 1 to 6, based on six aspects of the probiotic application (Supplementary Table S1): 1) content relating to the use of probiotics; 2) use under the guidance of experts; 3) doses and frequency of using probiotics; 4) change of symptoms after using probiotics; 5) side effects of probiotics; 6) precautions about using probiotics. The reliability assessment of videos was scored from 1 to 5 (Supplementary Table S2): 1) Is the video clear, concise, and understandable? 2) Are valid sources cited? 3) Is the content presented balanced and unbiased? 4) Are additional sources of content listed for reference? 5) Are areas of uncertainty mentioned?

Quality assessment of videos was scored using GQS criteria (Supplementary Table S3), and the information analysis in GQS was determined, considering whether they mentioned all the contents in the six domains described in Supplementary Table S1. The GQS was also scored from 1 to 5, classifying videos as “poor,” “generally poor,” “moderate,” “good,” and “excellent” accordingly. The positive/negative remarks percentage was calculated using the equation: Percentage = [the number of positive/negative remarks ÷ total remarks] × 100%.

**Fig. 1:**
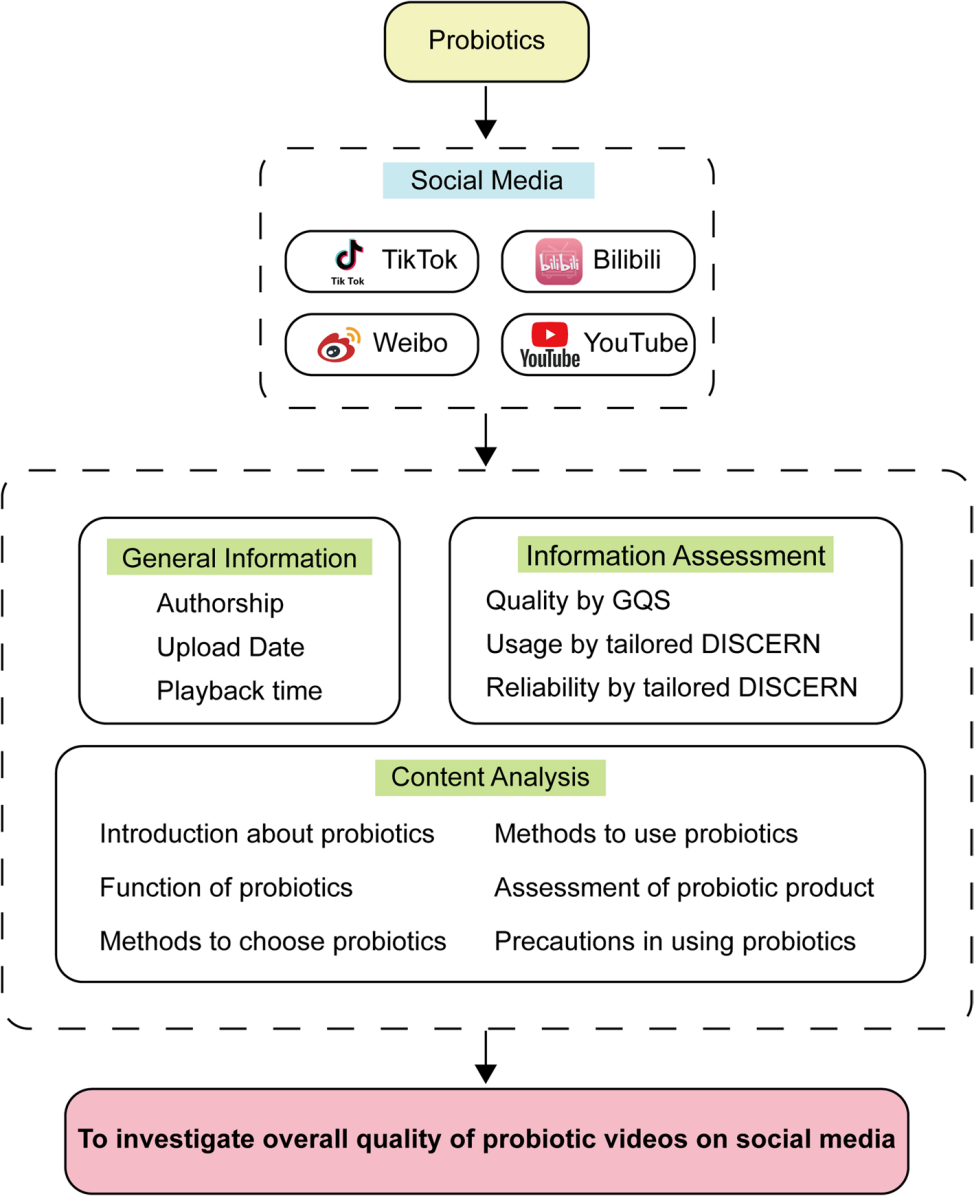
Framework of the study

The framework of the study was shown in Fig. 1.

### Statistical analysis

Data were analysed using R (version 4.2.1, *R* Foundation for Statistical Computing, Vienna, Austria). If the quantitative data conformed with normal distribution, then data were presented as mean ± standard deviation (‾x ± SD). If not, then data were presented as interquartile ranges. The Kruskal–Wallis rank sum test was employed to analyse the nonparametric data between groups. Categorical data were presented as frequency and ratios (%). If one or more of the cell counts in an R × C table is less than 5, Fisher exact tests were used to analyse the multi-sets of categorical variables. Otherwise, Chi-square tests were used. A *P* value < 0.05 was considered statistically significant. The adjusted *P* value using the Bonferroni method (for post hoc binary comparisons) was employed to evaluate the significance among multi-sets of data.

## Results

### Overview of the video filtering process

**Fig. 2:**
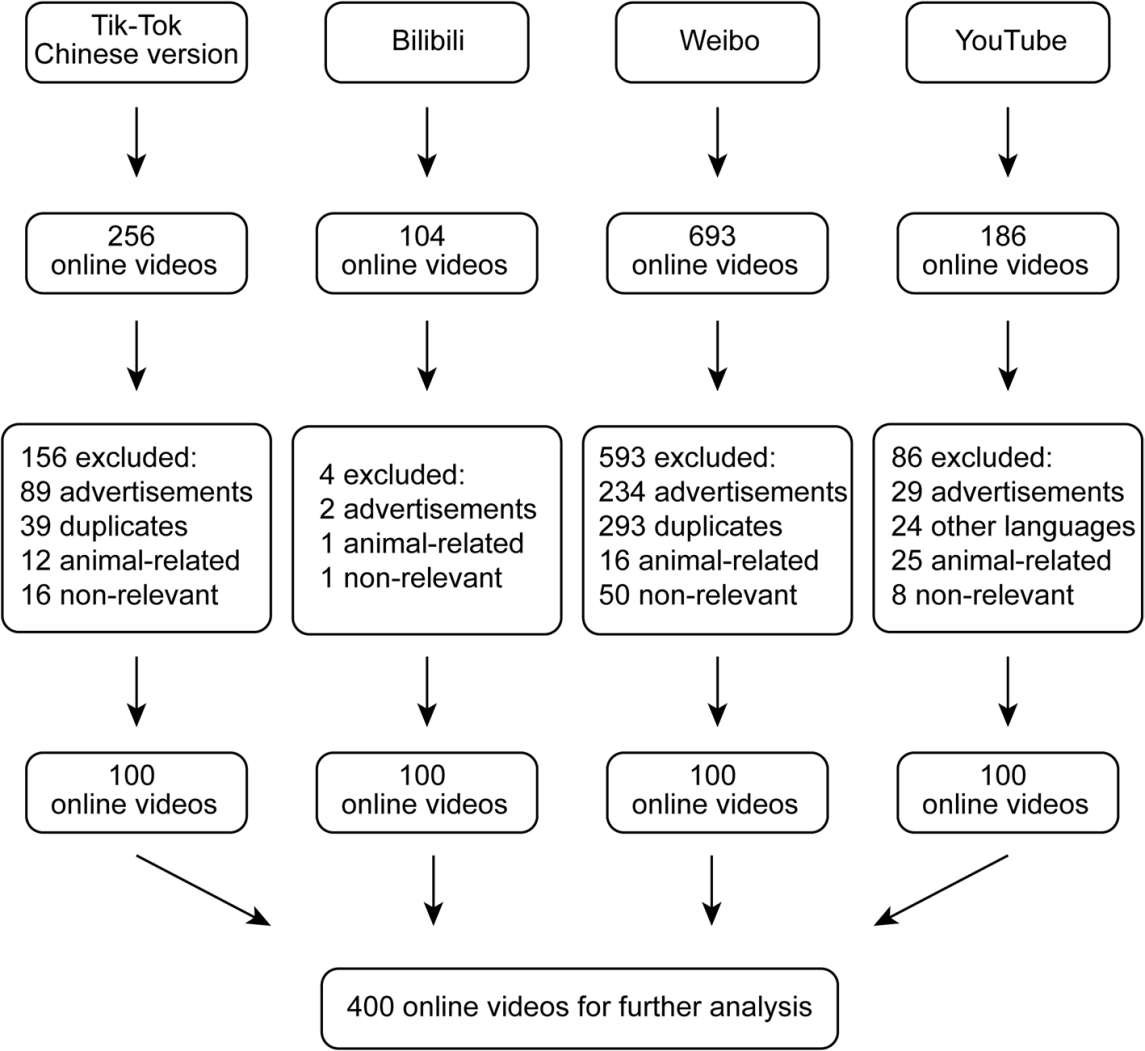
Flowchart of filtering probiotic videos for further analysis

One thousand two hundred thirty-nine probiotic-related videos were retrieved from the four platforms YouTube, TikTok, Bilibili, and Weibo. After removing 332 duplicated, 24 non-Chinese / non-English videos, 75 irrelevancy, 54 animal-related content, and 354 advertisements. As a result, four-hundred eligible videos were included for further analysis (Fig. 2).

### General information about the eligible videos

**Fig. 3:**
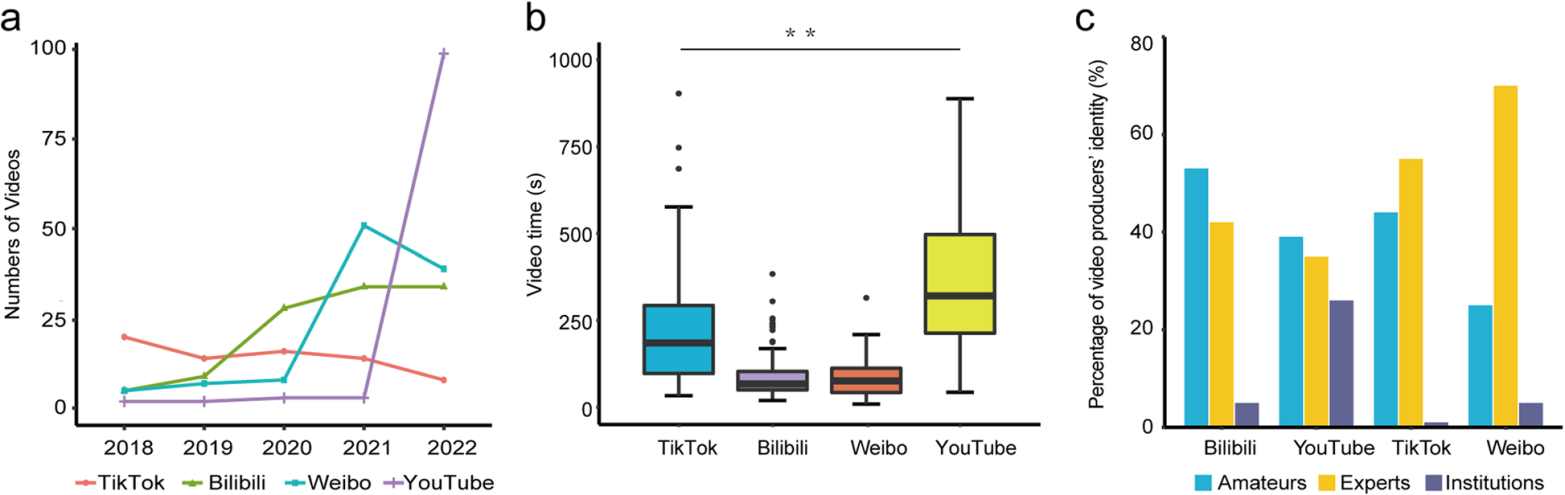
General information on probiotic-related videos sourced from the four video-sharing platforms. **a** A line chart shows 400 eligible probiotic-related videos released between 2018 and 2022 that met the inclusion criteria. **b** The playback time of probiotic-related videos on the four video-sharing platforms. **c** Bar chart about the percentage of video producers’ identity on the four video-sharing platforms

The uploaded videos were distributed between June 13^th^, 2008, to September 5^th^, 2022. Most of the included videos were from 24 in 2018 to 172 in 2022 (*n* = 361, 90.2%), with sporadic video uploads of 1 in 2008 to 7 in 2017 (*n* = 39, 9.8%). Most videos included on YouTube were from 2022 (97/400), while the other three platforms were mainly distributed between 2018 and 2022 (262/400) (Fig. 3a). The median playback time of videos is 129 (59.3, 257.8) seconds. The median playback time of videos on YouTube is longer than those on TikTok, Weibo and Bilibili (*P* < 0.001) (Fig. 3b). The identity distribution of video producers was predominantly experts (*n* = 202, 50.50%), followed by amateurs (*n* = 161, 40.25%) and health-related institutions (*n* = 37, 9.25%) (Table 1). There were more amateurs video producers on Bilibili, while more expert producers were on Weibo (*P* < 0.001) (Fig. 3c).

**Table 1:** Structural analysis of probiotic-related videos on the four video-sharing platforms

	Total	Tik-Tok	Bilibili	Weibo	YouTube	*P* value
Number	400	100	100	100	100	
Authorship						
Experts	202	55	42	70	35	< 0.001
Institutions	37	1	5	5	26	
Amateurs	161	44	53	25	39	
Video time (s)	129 (59.3,257.8)	186.5 (99.0,298.5)	67.0 (49.8,103.0)	77.5 (42.8,112.0)	326 (218.5,511.8)	< 0.001
Content category						
Introduction	57	12	17	10	18	< 0.001
Function	120	21	50	10	39	
Choose	81	19	5	45	12	
Usage	71	21	12	19	19	
Food/Products	44	14	12	11	7	
Precaution	27	13	4	5	5	
Producers’ Attitude						
Positive	323	65	86	77	95	< 0.001
Neutral	52	22	8	17	5	
Negative	25	13	6	6	0	
Audience’ Attitude						
Positive (%)	7.2 (3.6,12.5)	7.6 (4.2,11.2)	10.9 (5.5,20.6)	20.0 (10.0,24.7)	3.9 (2.2,6.5)	< 0.001
Negative (%)	1.8 (1.1,4.9)	1.5 (0.9,3.4)	6.6 (2.4,14.6)	22.2 (16.7,36.1)	1.3 (0.8,2.3)	< 0.001
^a^GQS score	3 (2,4)	3 (2,3)	3 (2,4)	3 (2, 3.3)	3 (2,4)	< 0.001
Usage score	1 (0,2)	1 (1,2)	1 (1,2)	0 (0,1)	1 (0,2)	< 0.001
Reliable score	2 (1,3)	1 (1,1)	2 (2,3)	1 (1,2)	3 (2,4)	< 0.001

^a^GQS Global quality scale

### Video content analysis

**Fig. 4:**
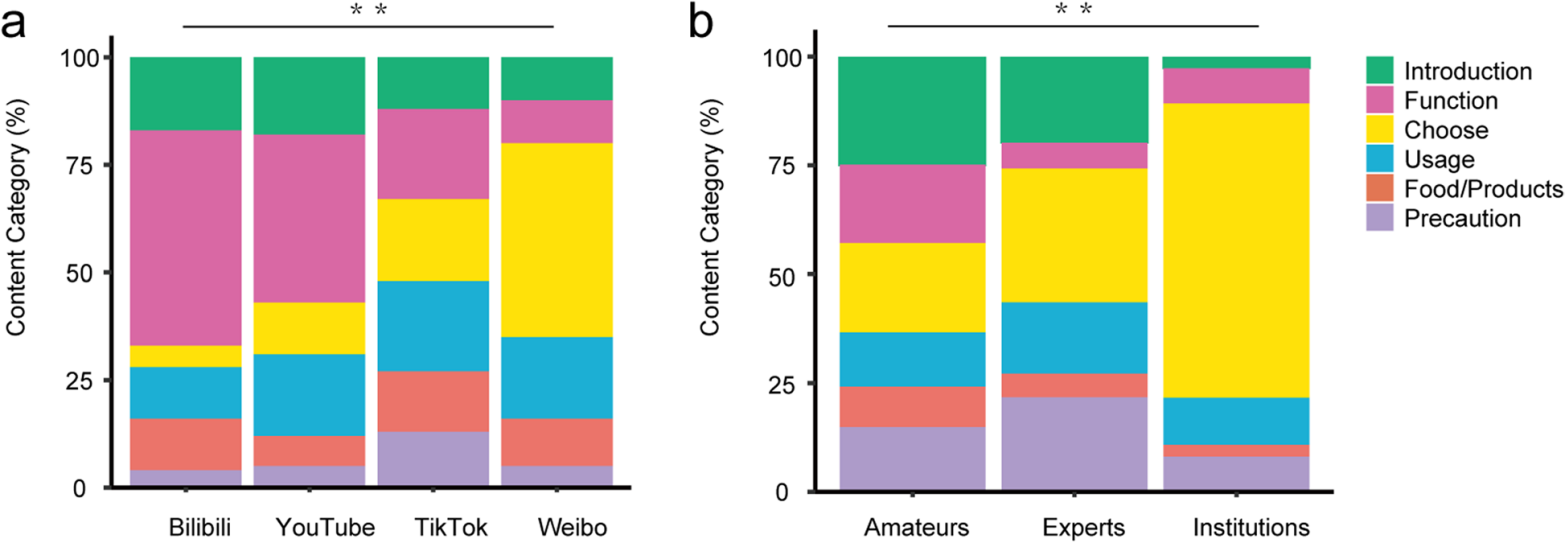
Characteristics of eligible probiotic-related videos on the four video-sharing platforms. **a** Stacking diagram showing the content category analysis of probiotic-related videos on the four video-sharing platforms. **b** Stacking diagram showing the content category analysis of probiotic-related videos released by three kinds of video producers

The content category of videos included discussions regarding the function of probiotics (*n* = 120, 30.00%), the way to choose suitable products (*n* = 81, 20.25%), the methods for taking probiotics (*n* = 71, 17.75%), introduction about probiotics (*n* = 57, 14.25%), assessment on the probiotic-related food or products (*n* = 44, 11%) and precautions in using probiotics (*n* = 27, 6.75%). There was no significant difference between Bilibili, YouTube, and Tik-Tok regarding the content category of videos. In contrast, each of them was significantly different from Weibo (*p* < 0.05) (Fig. 4a). Video producers on Bilibili and YouTube exerted more effort to explain probiotics’ functions. At the same time, those on Weibo mainly talk about choosing suitable probiotics-related products or medicine. Contingency table analysis between the video producers and content categories showed that experts emphasised the proper selection of probiotics (62/202), while amateurs focused on introducing the basic knowledge of probiotics (40/161) (*P* = 0.058) (Fig. 4b).

### Assessment of video information

**Fig. 5:**
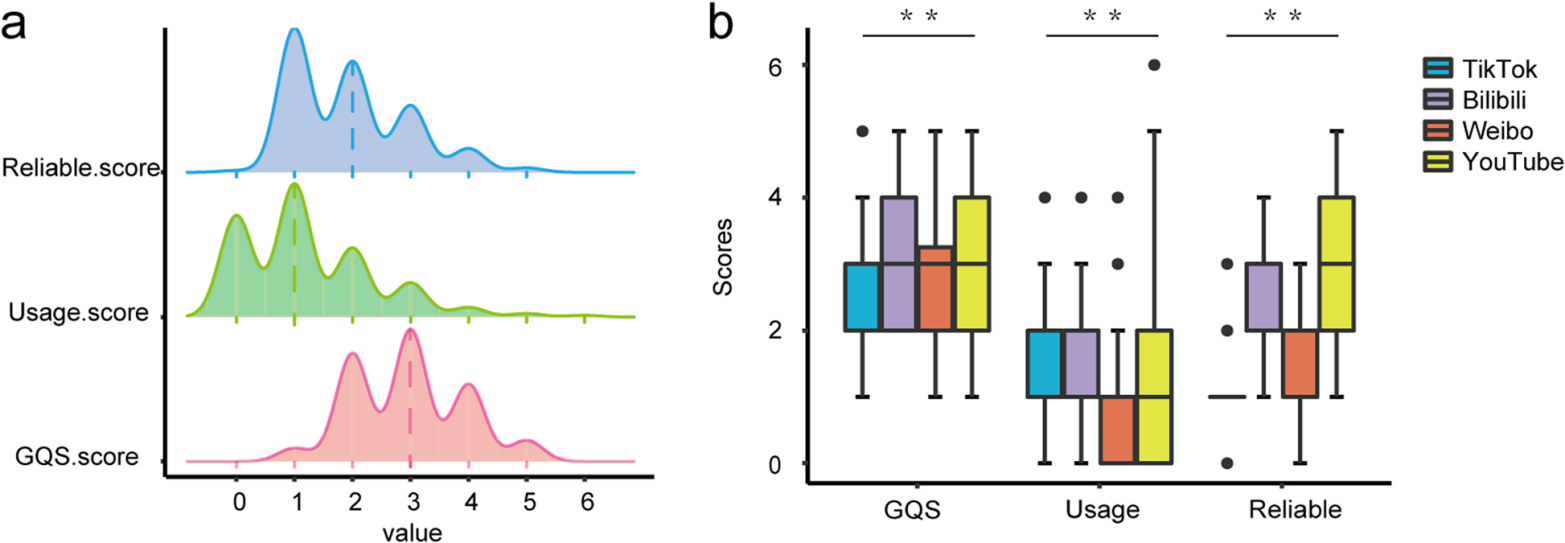
Assessment of video information. **a** Ridge plot showing the overall distribution of GQS, usage and reliability scores. **b** Assessment of probiotic-related videos on the four video-sharing platforms by GQS, usage and reliability scores. GQS: Global Quality Scale

Regarding the assessment of each video, the overall GQS quality of the uploaded videos is 3 (2, 4) points, while the usage and reliability of videos assessed by tailored DISCERN tool are 1 (0, 2) and 2 (1, 3) points (Fig. 5a). The GQS scores of videos on Bilibili and YouTube are better than those on TikTok (*P* < 0.05). The usage scores on TikTok, Bilibili and YouTube are better than those on Weibo (*P* < 0.001). The reliable score is YouTube > Bilibli > Weibo > TikTok (*P* < 0.05) (Fig. 5b). The assessment of each video concerning the usage, reliability, and quality scores shows that YouTube enlisted a better comprehensive level of videos than the other three platforms.

### Attitude analysis of video producers and audience

**Fig. 6:**
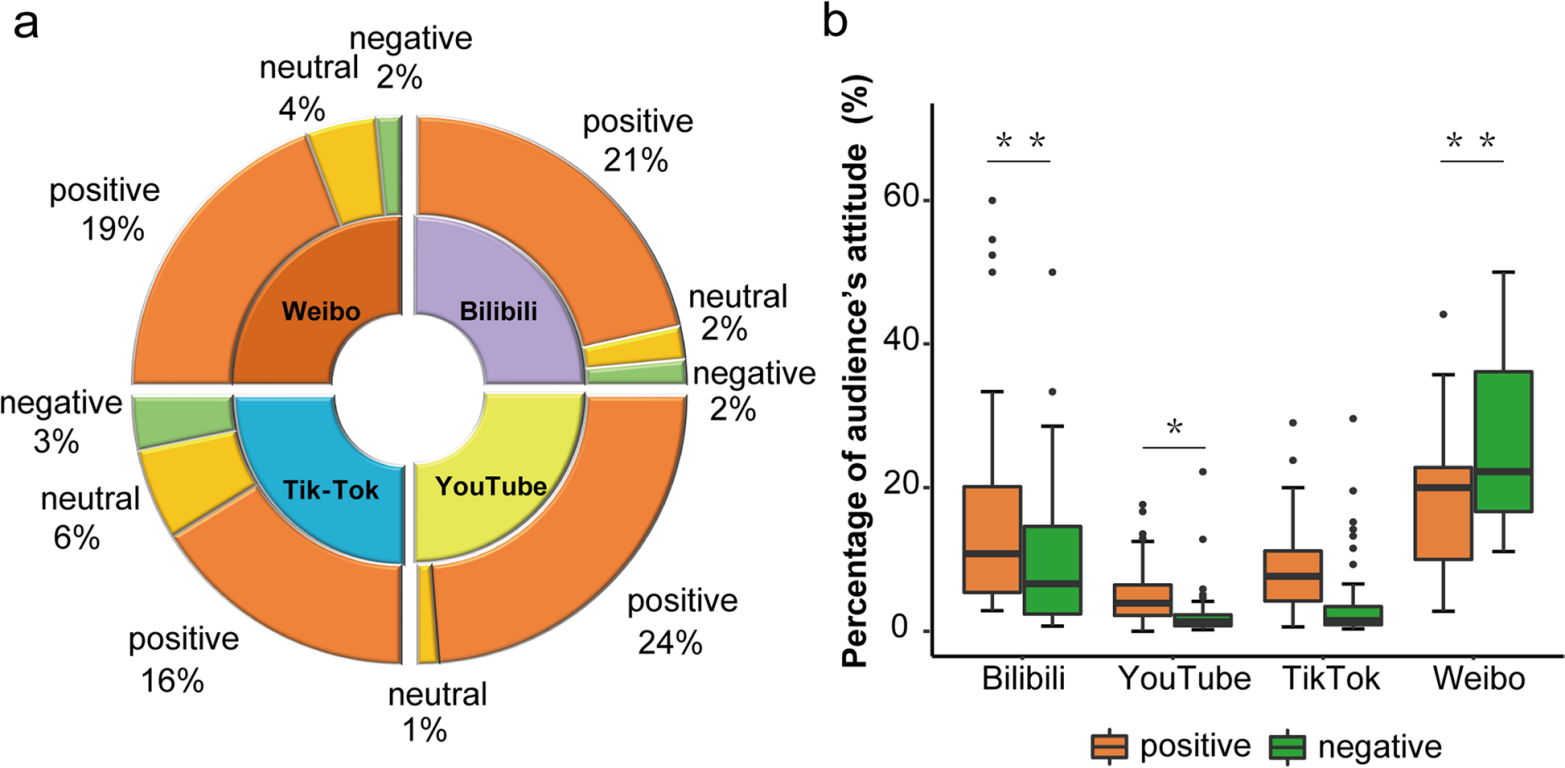
Attitude analysis of video producers and audience. **a** Sunburst chart detailing the attitude of video producers towards probiotics. **b** Box plot about the audience’s positive or negative attitude towards probiotics on the four video-sharing platforms

The attitude of video producers towards probiotics was primarily positive (*n* = 323, 80.75%), followed by neutral (*n* = 52, 13.00%) and negative (*n* = 25, 6.25%) on each social media platform (Fig. 6a). The audience’s attitude towards probiotics presented more positive 7.2% (3.6%, 12.5%) than negative 1.8% (1.1%, 4.9%) (*P* < 0.001) (Table 1). However, the audience on each platform had different attitudes, with Bilibili showing more positive while Weibo is more negative (*P* < 0.001) (Fig. 6b).

## Discussion

Probiotics are live microorganisms and confer a health benefit on the host when administered in appropriate amounts and strains [1]. Therefore, probiotics are gaining extensive attention, exploitation, and application in the health field. Probiotics may confer beneficial effects for diseases and symptoms such as constipation (*Bifidobacterium lactis*) [22], diarrhea (*Lactobacillus rhamnosus GG*) [23], and alleviate psychiatric disorders (*Bifidobacterium bifidum*) [24]. However, probiotics are not a panacea. Owing to the considerable heterogeneity in studies, there is limited evidence for probiotics in treating the disorders or diseases mentioned above. Also, the healthcare mechanisms of probiotics have not yet been fully elucidated and need further research. Probiotics may be associated with a higher risk of infection or mortality in critically ill patients, immune-compromised patients, infants, or neonates with very low birth weights [25–29]. Furthermore, clear guidelines on when and how to use probiotics for different disease conditions have not been established [30]. Considering the extensive application of probiotics in medicine, healthcare, and daily life, it is essential to popularise and make knowledge about probiotics (such as their therapeutic scope and side effects) widely available among the public.

Social media are potential tools to shorten health disparities in the current digital era. A study showed that 70—80% of Internet users seek health-related information online [31]. Previous studies showed that social media significantly increased knowledge of HPV vaccines and the audience’s willingness to accept the vaccine [32] and enhanced the self-care activities of patients with diabetes [33]. However, social media is a double-edged sword. Social media infodemic such as misinformation, disinformation, or false information can cause confusion and risk-taking behaviours that harm health [34]. Businesses and the pharmaceutical industry increasingly utilise the advantages of online media and over-advertise probiotic products. A previous study pointed out that the probiotic sector takes advantage of results obtained with a specific probiotic and then extends them to other fields, regardless of species/strain-specific effect, dose, duration intake, or mono- / multi-strain factors [35]. Also, lack of a peer-review system on social media platforms enables an explosion of unchecked information and the spread of misinformation [36].

This study included the four most popular video-sharing platforms domestically (TikTok, Bilibili, and Weibo) and abroad (YouTube). These social media platforms attract billions of active users with convenience, interactivity, and diversity [37–40]. The current study shows that videos on social media platforms publicise important information, including the function of probiotics, the way to choose suitable products, the methods for taking probiotics, and precautions in using probiotics. The availability of this information is essential to improve the public’s general understanding of probiotics. Half of the video producers are experts, indicating that medical practitioners actively publicise probiotics. However, the overall quality of uploaded videos about probiotics was moderate, while the usage and reliability were poor. The result indicated that social media should improve the comprehensive level of probiotic-related videos to benefit the public better. The attitude of video producers and audience presented more positive than negative attitudes towards probiotics, indicating that probiotics are popular in public and easy to accept as wholesome products.

The knowledge gap in the healthcare field makes it challenging for the public to discriminate good information from misinformation in the era of social media. Therefore, it is essential to popularise reliable information about probiotics, their therapeutic scope, and their side-effects to the public. First, regulators on social media platforms and other related regulatory departments should monitor health-related video content, resisting the broadcast of misleading videos on the Internet [41]. Second, platforms could optimise machine algorithms to promote evidence-based science websites to those seeking health-related information; they could also set a particular column to screen and spread authoritative information [42]. The more accurate and evidence-based information online videos provide, the more the public will benefit their health. In addition, related governments, professional organisations, and experts should actively rebut misinformation or producing high-quality health-related information on social media [43, 44]. Also, the public should selectively watch high-credibility videos as self-informed source and consult professionals for using probiotics to maintain health and treat illness.

Limitations of this study should be noted. As videos are constantly uploaded and deleted, only online videos available within a specific time frame have been analysed. Also, only videos in the Chinese and English languages are included in this study, which might omit some pertinent videos in other languages. These factors may lead to selection bias in this study.

## Conclusion

The current study provides valuable information for understanding the status quo of probiotic videos on social media platforms, which helps to enhance the quality of probiotic-related videos online and to promote the rational clinical usage of probiotics. The results show that videos on social media platforms publicise important information, including the concepts, usage, and precautions of probiotics to the public. Overall, however, the quality of uploaded videos about probiotics was unsatisfactory. More efforts are required to improve the higher-quality content of probiotic-related online videos to propagate better probiotic knowledge to the public.

## Supplementary Information

**Additional file 1: Table S1. **Usage assessment of video content. **Table S2.** Reliability assessment of videos. **Table S3.** Quality assessment of videos by GQS.

## Data Availability

All data that support the findings of this study are included in this manuscript and its supplementary information files.
