# Supplementary material for: Status quo of the public’s knowledge of probiotics based on video-sharing platforms
Source: BMC Public Health. 2023 Mar 28;23:574. doi: 10.1186/s12889-023-15456-7 (PMC10043532; doi:10.1186/s12889-023-15456-7)
Supplement: Supplementary file 1 — Additional file 1: Table S1. Usage assessment of video content. Table S2. Reliability assessment of videos. Table S3. Quality assessment of videos by GQS. [file 12889_2023_15456_MOESM1_ESM.pdf]

Supplements

Table S1 Usage assessment of video content

| Score Components | Score* |
|---|---|
| Content relating to the use of probiotics | 1 |
| Use under the guidance of experts | 1 |
| Doses and frequency of using probiotics | 1 |
| Change of symptoms after using probiotics | 1 |
| Side effects of probiotics | 1 |
| Precautions in using probiotics | 1 |
| Total score | 6 |

*Tally each single domain to get the total useful score, ranging from 0 to 6. 1 point for each aspect, the lowest score was 1 and a maximum score was 6.

Table S2 Reliability assessment of videos

| Reliability Score | Score* |
|---|---|
| 1. Is the video clear, concise, and understandable? | 1 |
| 2. Are valid sources cited? | 1 |
| 3. Is the content presented balanced and unbiased? | 1 |
| 4. Are additional sources of content listed for reference? | 1 |
| 5. Are areas of uncertainty mentioned? | 1 |

*Tally each single domain to get the total reliability score, ranging from 0 to 5. 1 point for each aspect, the lowest score was 1 and a maximum score was 5.

Table S3 Quality assessment of videos by GQS.

| GQS Definition | Score |
|---|---|
| Poor quality, poor flow of the video, most information missing, not at all useful for patients | 1 |
| Generally poor quality and poor flow, some information listed but many important topics missing, of very limited use to patients | 2 |
| Moderate quality, some important information is adequately discussed | 3 |
| Good quality good flow, most relevant information is covered, useful for patients | 4 |
| Excellent quality and flow, very useful for patients | 5 |

*GQS: Global Quality Scale. GQS was scored from 1 to 5, classified videos as “poor”, “generally poor”, “moderate”, “good”, and “excellent” accordingly.
